# Characterization and Applications of Nanoparticles Modified in-Flight with Silica or Silica-Organic Coatings

**DOI:** 10.3390/nano8070530

**Published:** 2018-07-14

**Authors:** Patrick Post, Lisa Wurlitzer, Wolfgang Maus-Friedrichs, Alfred P. Weber

**Affiliations:** 1 Institute of Particle Technology, Clausthal University of Technology, Leibnizstraße 19, 38678 Clausthal-Zellerfeld, Germany; patrick.post@tu-clausthal.de; 2 Institute of Energy Research and Physical Technologies, Clausthal University of Technology, Leibnizstraße 4, 38678 Clausthal-Zellerfeld, Germany; lisa.wurlitzer@tu-clausthal.de (L.W.); wolfgang.maus-friedrichs@tu-clausthal.de (W.M.-F.); 3 Clausthal Centre of Material Technology, Clausthal University of Technology, Agricolastraße 2, 38678 Clausthal-Zellerfeld, Germany

**Keywords:** core-shell, dielectric barrier discharge, in-flight nanoparticle coating, plasma enhanced chemical vapor deposition, silica, silica-organic

## Abstract

Nanoparticles are coated in-flight with a plasma-enhanced chemical vapor deposition (PECVD) process at ambient or elevated temperatures (up to 300 °C). Two silicon precursors, tetraethyl orthosilicate (TEOS) and hexamethyldisiloxane (HMDSO), are used to produce inorganic silica or silica-organic shells on Pt, Au and TiO_2_ particles. The morphology of the coated particles is examined with transmission electron microscopy (TEM) and the chemical composition is studied with Fourier-transform infrared spectroscopy (FTIR), Energy-dispersive X-ray spectroscopy (EDX) and X-ray photoelectron spectroscopy (XPS). It is found that both the precursor and certain core materials have an influence on the coating composition, while other parameters, such as the precursor concentration, aerosol residence time and temperature, influence the morphology, but hardly the chemical composition. The coated particles are used to demonstrate simple applications, such as the modification of the surface wettability of powders and the improvement or hampering of the photocatalytic activity of titania particles.

## 1. Introduction

Coated nanoparticles, often called core-shell particles, are used in numerous applications. Inorganic core particles combined with an inorganic shell of silica (SiO_2_) are one of the most studied core-shell combinations, since silica has interesting properties such as chemical inertness, optical transparency and an improved suspension stability [1,2]. For example, the photocatalytic activity of the UV-absorbing titania nanoparticles used in many sunscreens is disabled by a hermetic silica shell [2,3]. Depending on the coating process, films with different fractions of organic compounds can be produced as well, resulting in silica-organic (SiO*_x_*C*_y_*H*_z_*) coatings.

A significant portion of the common silica coating processes takes place in the liquid phase and often uses modified Stöber processes [1]. The sol-gel process can be used for the coating of particles with silica and silica-organic (organosilica) coatings for medical applications [4]. However, this method is not ideal from a practical standpoint, since many commercial nanoparticles with high production volumes are produced in the gas phase (e.g., flame synthesis of TiO_2_). Such particles would require two separate process environments (gas and liquid) for the production of core-shell structures. Furthermore, liquid phase processes are often time consuming and require many process steps to acquire by-product free dry powders. In contrast, gas phase processes generally involve less steps and lead to purer products [5].

The majority of gas phase processes for silica or silica-organic coatings use a form of chemical vapor deposition (CVD), where a precursor reacts to the desired coating material. Common aerosol coating techniques include flame synthesis [6,7], where particle production and coating can be achieved in one reactor, atomic layer deposition [3,8], which allows the formation of very small and defined films, and hot-wall reactors [9], where the precursor is converted through thermal energy, without flame. However, these techniques, especially flame synthesis and hot-wall reactors, require high temperatures for the coating formation and are hence not suitable for thermally sensitive core materials. Others, such as atomic layer deposition, are difficult to transfer to a continuous mode of operation, due to complex requirements on the coating environment. A method to continuously coat particles with silica at low temperatures is the use of a non-thermal plasma, such as a dielectric barrier discharge (DBD), which provides a chemically reactive environment [10,11,12]. However, the direct introduction of the particles and precursor into the discharge causes high losses and results in a reduced long-term stability in continuous operation mode, due to material deposition on the dielectrics.

This problem can be avoided by coating the particles in the post-discharge environment, which was shown for silica coatings with TEOS as the precursor and allowed the coating of temperature sensitive materials such as certain salts and polymers [13,14]. Bulk substrates, such as Si-wafers, are often coated in a heated post-discharge environment with ozone and the coating precursor. Ozone will easily form highly reactive oxygen radicals through thermal decomposition and so reduces the required process temperature significantly to a few hundred Degree Celsius [15,16].

In this work, such a post-discharge process will be used for the coating of different core particle materials with silica or silica-organic shells. Two process variants will be used, one with the coating environment at ambient temperature and another at elevated temperatures of up to 300 °C. It will be shown that the combination of precursor, particle material and temperature influence the morphology of the coatings. The focus of this manuscript will be on the study of the chemical composition of the coatings with FTIR, EDX and XPS. Finally, examples for possible applications derived from the chemical composition and morphology of the coatings will be demonstrated. While the feasibility and some features of the coating process will be shown here, the detailed understanding of the shell formation is beyond the scope of this article.

## 2. Materials and Methods

**Figure 1:**
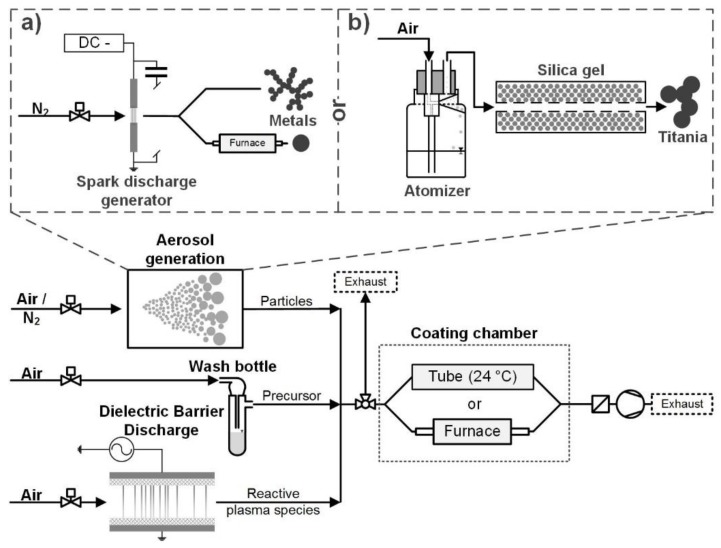
Experimental setup. Two different aerosol generation methods were used for the core particles: a spark discharge generator for metals (Pt or Au) (**a**) and an atomizer for titania (**b**). The coating precursors were TEOS and HMDSO.

The particles were produced and coated in the gas phase in a continuous aerosol process at ambient pressure. The precursor vapor was mixed with the aerosol particles and the coating formation was facilitated by reactive species from the exhaust of a dielectric barrier discharge reactor. The process can be described as plasma-enhanced chemical vapor deposition (PECVD). Figure 1 shows an overview of the experimental setup. The following sections will describe the individual setup parts in more detail.

### 2.1. Aerosol Generation

Different particle materials were used as cores for the coating. Metal particles (Au and Pt) were produced in a spark discharge generator (SDG, Figure 1a). A SDG produces fractal nanoparticle agglomerates of small primary particles (single digit nm) by ablation of electrodes with an electrical spark [17]. The carrier gas was 1 L/min nitrogen (N_2_ 99.999%). The spark discharge was operated by charging a capacitor of 24 nF with a current of 0.8 mA until a spark was created between the electrodes. The cylindrical electrodes were 3 mm in diameter and positioned with about 2 mm distance to each other. The Pt and Au electrodes had purities of 99.99% and 99.9%, respectively. The metal agglomerates could be introduced into a tube furnace to sinter them to sphere-like particles, which required temperatures of 800 °C for Au and 1000 °C for Pt (6 s and 5 s residence time, respectively). 

Alternatively, commercial titania particles were atomized from an aqueous suspension (Figure 1b). The suspension had a concentration of 1 mg/ml TiO_2_ (Degussa/Evonik Aeroxide TiO_2_ P25). P25 is a commercially available mixture of rutile and anatase nanoparticles, produced by flame synthesis, which is used for its high photocatalytic activity [18]. The suspension was sprayed with an atomizer (Topas GmbH, Dresden, Germany, ATM 220) and the droplets were dried in a silica gel denuder, leaving the P25 aerosol. The atomizer was operated with 1.5 bar filtered pressurized air, which resulted in an aerosol flow of 1 L·min^−1^.

### 2.2. Particle Coating

Two precursors were used in this work: tetraethyl orthosilicate (TEOS, SiC_8_H_20_O_4_) and hexamethyldisiloxane (HMDSO, Si_2_C_6_H_18_O). For the particle coating, the precursor was kept in a small washing bottle, consisting of a modified test tube with a gas inlet and outlet (Figure 1). A small carrier gas flow of air (mL/min) was saturated with the precursor and mixed with the core particles in a simple T-piece.

The reactive species necessary to initiate the coating reactions were produced in a DBD plasma. The symmetrical self-built plate-to-plate reactor had a discharge area of 729 mm² with a discharge gap of 1 mm. Two alumina dielectrics of 2 mm thickness between the two electrodes were used. The discharge was operated in 2 L·min^−1^ filtered pressurized air and the resulting gas mixture introduced into the coating system. The discharge power was kept constant at about 11 W (measured by Lissajous figure), resulting in a temperature of about 50 °C on the outer electrode surfaces.

After the mixing of particles, precursor and plasma species, the coating formation starts. However, there are two options for how to proceed. The first is the coating reaction at ambient temperature. This is only possible with TEOS and requires residence times in the range of minutes for the formation of solid coatings. During the coating, the mixture is pumped through a 4.2 L quartz glass tube. The second option is to heat the mixture in a tube furnace up to about 300 °C. This is possible for TEOS and HMDSO and results in much faster coating kinetics within seconds. For both variants, the aerosol residence time of the particles between mixing and sampling is controlled by the volume flow through the coating chamber (glass tube or furnace). The aerosol residence time provided is an average time (volume/volume flow), since a laminar flow is expected in the coating chamber (Re << 2300). The excess aerosol that is not pumped through the coating chamber leaves the system to the exhaust.

### 2.3. Coating Analysis

For transmission electron microscopy (TEM, JEOL (Germany) GmbH, JEM 2100, Eching, Germany), particles were sampled either directly from the aerosol or from samples collected on a filter. Energy-dispersive X-ray spectroscopy (EDX, OXFORD Instruments GmbH, X-Max^N^ 80 T, Wiesbaden, Germany) was performed in the TEM system. For Fourier-transform infrared (FTIR, Bruker Optik GmbH, Tensor 27, Ettlingen, Germany) spectroscopy and X-ray photoelectron spectroscopy (XPS, PREVAC sp.zo.o, Poland), particles were collected on a polycarbonate membrane filter (0.2 µm pores). For FTIR measurements, the powder was removed from the filter and pressed with KBr powder. Since the available powder mass was low, due to the lab-scale particle synthesis methods, a collection time of about 7 h in the system was required. XPS measurements were done directly on the membranes. To reduce charging effects, the membranes were modified with a Cr coating of a few nm thickness by sputtering. The XPS measurements were carried out in an ultrahigh vacuum (UHV) chamber, with a base pressure below 3 × 10^−10^ mbar. The apparatus setup is in detail described previously [19,20,21]. The electron spectroscopy was performed by a commercial X-ray source (RS40B1, Prevac, Rogów, Poland) with a hemispheric analyzer with a resolution of 1.1 eV under an angle of 10° to the surface normal for emitted electrons. It exhibits a full width at half maximum (FWHM) of 1.9 eV for clean graphite. The X-ray photons hit the sample surface under an angle of 80° to the surface normal irradiating the whole sample. The presented results were measured with Al Kα line with an energy of 1486.6 eV and Mg Kα line with an energy of 1253.6 eV. All XP spectra are displayed as a function of binding energy with regard to the Fermi level. For quantitative XPS analysis, the areas of the photoelectron peaks are calculated after Shirley background correction [22]. Peak fitting was enforced by Gauss-type profiles with CasaXPS software (version 2.3.16 Pre-rel 1.4, CasaXPS Ltd., Teignmouth, United Kingdom). The photoelectric cross-sections were calculated by Scofield [23] and the asymmetric factors by Yeh and Lindau [24].

### 2.4. Photocatalytic Characterization

The degradation of methylene blue (MB) was used to quantify the photocatalytic activity of silica-coated P25 titania particles under UV radiation. The collected particle mass, sampled from the system with a filter, was typically below 1 mg. The coated particles were mixed with 10 mL aqueous MB solution (72 µmol/l) in a small petri dish. As reference, unmodified P25 of equal mass was put into a second dish with another 10 mL of the same solution. The dishes had a diameter of 53 mm. Both dishes were placed together in an ultrasonic bath for 3 min and then under an UV lamp (Philips TUV 4W, λ = 254 nm) for 1 h in an otherwise dark environment. The dishes were positioned at fixed locations close together and about 7 cm under the lamp, so that all samples and references experienced equal radiation. The suspensions were subsequently centrifuged and the particle-free supernatant measured by light absorption at 690 nm (Cole-Parmer, Staffordshire, United Kingdom, JENWAY Genova). It should be noted that the maximum absorption wavelength of MB might shift during photocatalysis, which could potentially influence the results. Three transmission measurement values were acquired per sample: the original MB solution, the solution after the reaction with the coated particles and the solution after the reaction with the reference particles. Since the transmission is related to the concentration of MB in the solution, the conversion of MB on coated and reference particles could be calculated from these values.

## 3. Results and Discussion

### 3.1. Coating Morphology

**Figure 2:**
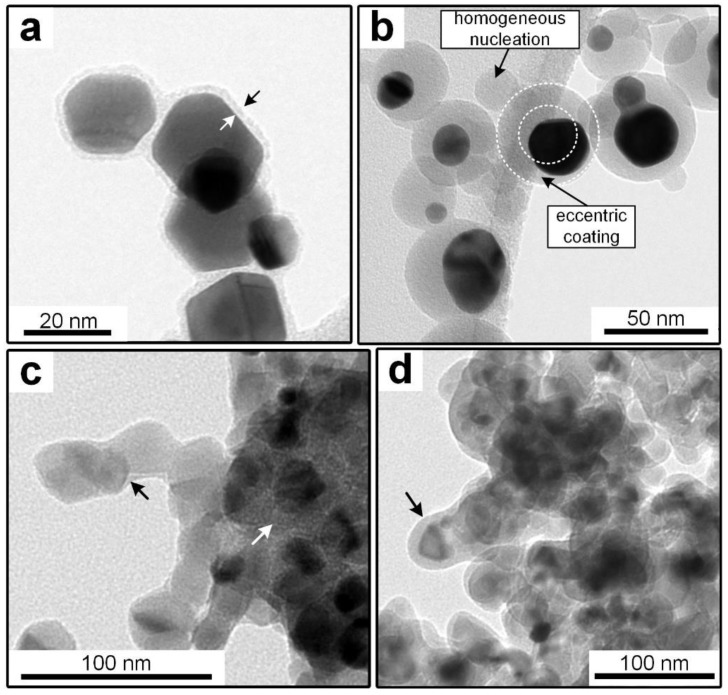
TEM micrographs of particles coated in the process. (**a**) Au, coated at 200 °C with TEOS; (**b**) Au, coated at 250 °C with HMDSO; (**c**) TiO_2_; coated at 24 °C with TEOS; (**d**) TiO_2_, coated at 200 °C with TEOS.

Different combinations of core and shell materials can be produced with the post-discharge coating process. Previously published work focused on silica coating with TEOS as the precursor at ambient temperature, since it allows the formation of coatings on thermally unstable materials. Examples of core particles that were previously used include metals and metal oxides from a SDG such as platinum, gold, copper oxide, titania, nickel oxide and iron oxide, as well as salts and polymers [13,14]. For all studied cases, TEM images showed coatings on all particles with no uncoated core particles found on the samples. However, in the case of complex agglomerates, some primary particles might exhibit uncoated surfaces, as is the case for titania particles (Figure 2c). The distribution of the coating thickness in a sample was studied before for the idealized system of homogeneously coated spherical gold particles [14]. It was found that, regardless of core particle size and coating thickness, the coating thickness on individual particles was within about 20% of the sample average.

While the mechanisms leading to the coatings are not completely understood, primarily due to the complexities of plasma chemistry, previous observations indicated reactions in the gas phase and on core particle surfaces. The gas phase reactions result in the formation of silica particles if no core particles are introduced into the system. It is simplifying assumed that a liquid intermediate product is formed in a first step, which covers the core particles (heterogeneous condensation) and slowly solidifies to the finished coating in a second step, probably by the proceeding polymerization [13]. Due to the big influence of gas phase reactions, the core material seems to play only a secondary role. In most cases, this seems limited to the wettability of the core material with the coating material intermediate (compare TEOS vs. HMDSO on metal, Figure 2a,b). However, certain materials might directly interact with the coating as shown for titania in Section 3.2. In contrast, the total surface area of the core particle aerosol, given by the dimensions of individual particles and the number concentration, directly influences the coating thickness as shown before [14]. It was found that an increase in total core particle surface area decreased the coating thickness when all other parameters were constant.

The main disadvantage of the process variant with TEOS at ambient temperature is the relatively long aerosol residence time of a few minutes (typically around 4 min). By increasing the temperature in the post-plasma environment to between 100 to 300 °C, the required time can be significantly lowered to a few seconds. This is believed to be related to the increased formation rate of highly reactive oxygen radicals from the thermal decomposition of ozone [16]. As shown in Figure 2a, the coating with TEOS at elevated temperatures allows the formation of homogenous coatings, which are morphologically similar to those acquired at ambient temperature. In the micrographs, the darkest parts are the core particles (nearly black for Pt or Au, dark gray for titania) and the lighter gray borders around them are the coating (marked with arrows in a). Other light gray areas belong to the background (e.g., TEM grid).

The elevated temperatures and the resulting increased reactivity also allow the use of HMDSO as an alternative precursor, which does not form coatings at ambient temperature. However, as shown in Figure 2b, the coatings acquired on the sintered gold particles were significantly less homogeneous, as evidenced by a high degree of eccentric coatings and homogeneous nucleation. This indicates a decreased wettability of the coating or its intermediates on the core particles. Since pure gold is typically hydrophilic [25], this might point to a hydrophobic character of the coating material.

Another core material used in this work is commercial P25 titania. These well-known nanoparticles are considered by many to be the gold standard of photocatalytic activity. The primary particles have a mean diameter of approximately 21 nm and exist in aggregates of several hundred nm in size [18]. Figure 2c,d show the comparison of the TEOS coatings at 24 °C (c) and 200 °C (d) on P25. While the coating morphology on the gold particles was similar for the different temperatures, the shells on P25 showed differences on the TEM micrographs. Even though the TEOS concentration was constant for both samples (9 ppmv), the coatings at 200 °C were thicker, which could be related to a higher conversion rate. Since the coating thickness is hard to define on inhomogeneously coated particles, EDX measurements were done to quantify the coating amount on the samples. The results are shown later in Figure 11, where the error bars in the x axis describe the standard deviations of the atomic ratio of Si to Ti atoms. The relative error was similar for all samples at around ± 20%. More interesting is the location of the coating material on the aggregates. While the coating is distributed relatively homogeneously over all surfaces at 200 °C, most of the material at 24 °C was collected in the inner parts of the structures (see white arrow in Figure 2c). This might be related to the speed at which the coatings solidified on the particles, since capillary forces might move non-hardened liquid intermediates into the inner particle structures.

### 3.2. Coating Composition with Different Precursors and Process Conditions

#### 3.2.1. TEOS at Ambient Temperature

**Figure 3:**
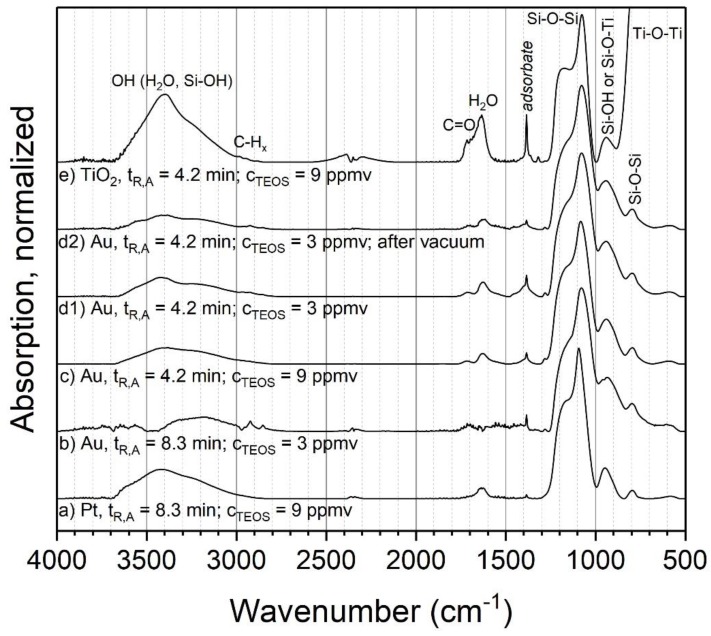
FTIR spectra of coatings with TEOS as the precursor at ambient temperature. *t*_R,A_:aerosol residence time, C_TEOS_:initial TEOS concentration. The spectrum c was published before and is reproduced with permission from the copyright holder Wiley-VCH Verlag GmbH & Co. KGaA [14].

The chemical composition of the produced coatings was analyzed with FTIR, EDX and XPS. The influence of different process parameter combinations on the chemical composition of the coatings with TEOS was first examined for ambient temperature (24 °C). Figure 3 shows the FTIR spectra for different powders coated at ambient temperature. Four different combinations of aerosol residence time and initial TEOS concentration were analyzed for metal particles (a–d1) and one of them was repeated and stored in a low-pressure environment of 10^−7^ Pa for 2 h (prior to the measurement) (d2). Other parameters, such as the discharge power and gas composition, were kept constant between the experiments. Spark discharge-generated Au (b–d) and Pt (a) particles were used as the cores without sintering. Additionally, P25 titania was atomized and coated (e). The spectra were normalized to the height of the respectively highest Si-O-Si peak to remove the influence of the measured powder mass and coating thickness to make the spectra more easily comparable. Since metal particles were used as the cores, which show no absorption bands in FTIR, all observed signals in Figure 3a to d2 can be assigned to the coating.

For the metal particles, it was found that the four parameter combinations resulted in very similar compositions (a–d1). The dominant peak at about 1080 cm^−1^ with a shoulder at about 1170 cm^−1^ can be assigned to the asymmetric stretching of siloxane (Si–O–Si). The associated bending vibration is present at about 800 cm^−1^ [26]. Distinctive Si-OH bending can be observed at about 950 cm^−1^ and further OH stretching modes are found around 3400 cm^−1^ that originate from Si–OH and adsorbed water. The presence of H_2_O on or in the sample is further corroborated by the OH bending of water around 1630 cm^−1^. The biggest differences between the samples are the peaks at about 1720 cm^−1^ and 1380 cm^−1^, the first of which can be assigned to C=O. The assignment of the second peak is not entirely clear, but it could correspond to methyl groups. The position of the C=O peak indicates aldehydes or esters.

One of the key questions concerning the chemical composition of silica coatings produced with PECVD from silicon organic precursors such as TEOS is the fraction of organic compounds and other by-products in the coating, since this influences the properties and possible applications of the product. While the results show some organic compounds (C=O and possibly methyl), their ratio in the total coating mass seems low (although quantitative information is not readily available from these measurements). Furthermore, while methyl groups might be expected in a silica-organic coating, C=O is probably not and rather indicates adsorbed by-products of the PECVD reactions. Such by-products should be relatively easy to remove either by heating the sample or by lowering the surrounding gas pressure. Since subsequent heating of the sample might influence the coating, one of the parameter combinations (d2, *t*_R_ = 4.2 min and C_TEOS_ = 3 ppmv) was repeated and the sample was put into a low-pressure environment to remove adsorbed compounds. The results show that the aforementioned species were indeed reduced in comparison to the original sample, although they did not disappear completely. This indicates that the coating itself seems to be mostly pure inorganic silica. This is further corroborated by EDX measurements, which were done for samples b, c and d1. Sample b showed Si to O atomic ratios of 1.99 ± 0.06, c 1.99 ± 0.1 and d1 2.05 ± 0.15, respectively. These results are close to the expected ratio of 2 in stoichiometric silica.

The titania sample (Figure 3e) shows a spectrum with similar structures as those found for the coated Au and Pt particles. In comparison to the metal particles, the biggest difference is the Ti–O–Ti asymmetric stretching vibration at about 550 cm^−1^, originating from the core particles. Otherwise, the sample shows similar, but larger, signals as the metal particles at about 1720 cm^−1^ and 1380 cm^−1^, which presumably also correlate with adsorbed species. Another potential difference is the peak at about 930 cm^−1^. While this could be assigned to Si–OH, as in the case of coated metal particles, it could also correspond to Ti–O–Si bonds on the interface of core and shell. However, a second band sometimes associated with Ti–O–Si around 1538 cm^−1^ is not present in this sample [27].

**Figure 4:**
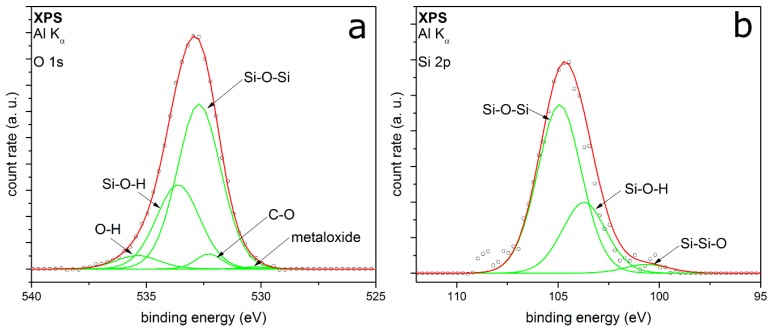
XPS detail spectra of (**a**) O 1s and (**b**) Si 2p of Au metal particles coated with TEOS as precursor.

The XPS detail spectra of TEOS-coated metal particles are shown in Figure 4. The O 1s peak (Figure 4a) is fitted with five peaks, which can be well adapted. The peaks associate the identified peaks in the FTIR spectrum (Figure 3). The two peaks with the highest relative intensities are at binding energies (E_B_) at 532.7 eV and 533.2 eV, respectively. These two peaks correspond to Si–O–Si and Si–OH [28,29,30,31], showing a silicon oxide coating on the surface with silanol groups in addition. It can be assumed that the Si-OH should be at the outer atomic layer of the SiO*_x_* coating [32]. A small amount of by-products with C–O bonding at E_B_ at 532.3 eV can be identified, which could be also C=O or C–O–H bonding [33]. This cannot be clearly distinguished because the *E*_B_ of the different species are intertwined. This supports the suspicion of a small amount of organic by-products, which remain in ultrahigh vacuum in the coated shell. The highest E_B_ corresponds to chemisorbed OH groups, resulting from small amounts of water contaminations [34]. The minor peak at 530.3 eV E_B_ can be assigned to metal oxides, which belong to molybdenum oxide. It is a generally detected species in our measurements because of the metal of the sample holder. The Si 2p peak shown in Figure 4b can be fitted with three peaks quite well. The peaks at 104.9 eV and 103.7 eV associate to the Si–O–Si and Si–OH from the O 1s spectrum. The positions are shifted to higher binding energies because of a partial charge of the deposited coating. Barr et al. show a shift of the silicon oxide part by charging to higher binding energies, while the non-oxidized fraction shows no shift [35]. The small peak at 100.6 eV could correspond to Si–Si–O or Si–C [33], which cannot be clearly distinguished.

**Figure 5:**
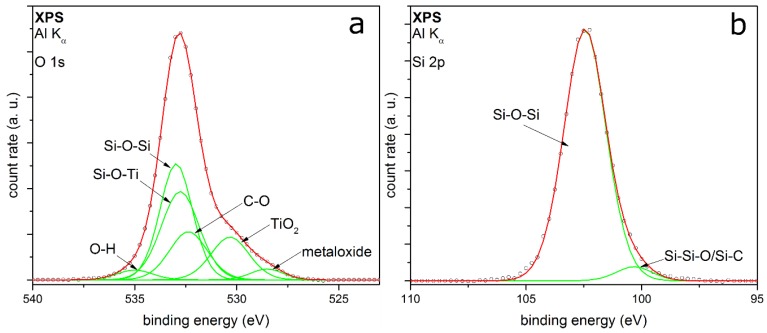
XPS detail spectra of (**a**) O 1s and (**b**) Si 2p of titania particles coated with TEOS as precursor.

Figure 5 shows the XPS detail spectra of TEOS-coated titania particles. The O 1s detail peak (Figure 5a) can be fitted here with six peaks. The peaks at E_B_ at 528.5 eV and 530.3 eV correspond to metal oxides like molybdenum oxide [36] and titanium dioxide [37,38], respectively. TiO_2_ is detected, because it is the core of the particles. A reason for the higher relative intensity of the core material than for the metal particles measurements could be a coating thickness smaller than 100 monolayers, which corresponds to a layer thickness of approx. 10 nm [39]. Another explanation could be an incoherent coating on the titania particles, which is also supported by the TEM measurements. These XPS results substantiate the formation of mixed oxides from silicon and titanium, which have been discussed for the FTIR results earlier in this section. Chinh et al. recently reported comparable mixed oxides produced by a sol-gel synthesis method of nanoparticles from tetrabutyl orthotitanate (TBOT) and TEOS [40]. Furthermore, there are peaks in the O 1s spectrum at 532.4 eV, 533.0 eV and 535.0 eV. These peaks can be assigned again to the C–O and the Si–O–Si bonding [28,33,40]. The highest binding energy is reported to be chemisorbed OH groups, same as the previous measurements. The peak at 532.8 eV belongs potentially to the Si–O–Ti mixed oxide, which has been mentioned before. The Si 2p detail peak (Figure 5b) can be fitted with two peaks at binding energies of 102.5 eV and 100.7 eV, mostly depending on Si–O–Si from silicon dioxide coating [28,35]. In contrast to the metal particles, no Si–OH bonding can be identified in both spectra. The fitting results from the XPS measurements for TEOS-coated particles are summarized in Table 1.

The XPS measurements show silica-coated particles beside the core material. The degree of organic compounds left in the coating is very small. Hence, the organic precursor reacts for the most part to silica. The produced by-products can be removed for the most part by heating or in vacuum. Using titania as core material, there is no Si-OH detectable in XPS; thus, it can be assumed that the metal oxide core promotes the formation of silicon oxide. Because of the absent Si–OH peak in the fitting of coated titania, it can be assumed that the FTIR peak at 930 cm^−1^ (Figure 3) can be assigned to Ti–O–Si and not to Si–OH, although the second FTIR band is not visible. Furthermore, this mixed oxide is also shown in the XPS O 1s spectrum (Figure 5a). The Ti–O–Si bond will be at the interface between the shell and the core material. These results indicate that the core material TiO_2_ directly interacts with the formation of the silica coatings. The possible presence of Ti–O–Si shows chemical bonding between core and coating, which was not observed for the Au and Pt particles, where the interaction seems to be purely physical. This seems to be coupled with the number of Si–OH groups found in the coating, which might indicate their involvement in the formation of the chemical bonding with titania. The mechanisms leading to this behavior are unknown at this point, but could involve different reactive species from the air discharge as well as coating intermediates and specific titania surface properties.

The results for the TEOS-coated particles at ambient temperature indicate that the influence of the initial TEOS concentration and the aerosol residence time on the coating composition is rather small. Hence, this process variant with TEOS at 24 °C allows the control of the coating thickness on particles as shown before [13,14], while maintaining an inorganic silica composition.

#### 3.2.2. TEOS at Elevated Temperatures

**Figure 6:**
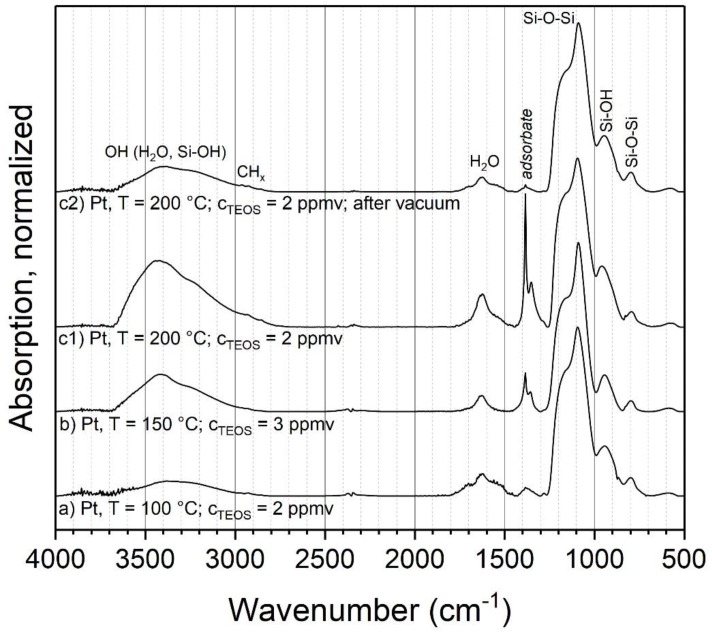
FTIR spectra of coatings with TEOS as the precursor at elevated temperatures.

Another parameter that might influence the coating composition is the temperature of the post-discharge environment. For this experiment, three different parameter combinations of temperatures and initial TEOS concentrations with metal particles were used. The volume flow controlling the residence time was kept constant, which resulted in a theoretical aerosol residence time in the tube furnace of 6 s at 24 °C. Due to the increase in gas volume during heating, the actual residence time in the furnace decreased somewhat with higher temperatures. Sintered particles were used as cores, to prevent further restructuring or sintering of particles during the coating process. Figure 6 shows the resulting FTIR spectra for the parameter combinations a–c1 and the repeated and vacuum outgassed sample c2, analogous to the experiments at ambient temperature.

For the most part, the same modes as in the experiments at ambient temperature are observed at elevated temperatures, such as Si–O–Si, Si–OH and adsorbed water. However, significant changes are visible in the peaks assigned to organic compounds. The C=O peak at 1720 cm^−1^ is missing, while the band at 1380 cm^−1^ has a much higher relative intensity and shows a second, smaller peak at slightly lower wave numbers. Furthermore, the band at 1380 cm^−1^ increases significantly with the furnace temperature. As discussed above, this could be related to actual parts of the coating or adsorbed by-products. Hence, sample c1 was repeated and introduced into vacuum, to remove eventual adsorbates (sample c2). The spectrum of this experiment shows a virtually complete removal of these bands, which clearly indicates adsorbed species. Hence, the coatings with TEOS at elevated temperatures seem to consist of inorganic silica as well. The larger amount of adsorbed species compared to 24 °C could be related to the much faster coating formation, possibly resulting in the entrapment of vapor in the coating and higher local concentrations of by-products near the particle surfaces.

#### 3.2.3. HMDSO

HMDSO was used as the second precursor in this work and required elevated temperatures for coating. The precursor molecule has direct bonds between the Si and C atoms, in contrast to TEOS, in which an oxygen atom is situated between Si and C. Hence, the finished coatings with HMDSO might contain a higher degree of organic components, even though HMDSO is also known as a precursor for inorganic silica coatings, e.g., in flame spray pyrolysis [6].

**Figure 7:**
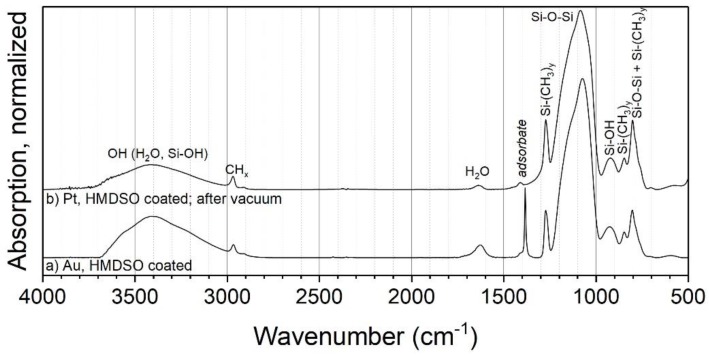
FTIR spectra of coatings on metal particles with HMDSO as the precursor at 250 °C.

Coatings with HMDSO were acquired in the PECVD process on metal agglomerates at 250 °C (Figure 7). The spectra exhibit similar peaks as the coatings with TEOS, such as Si-O-Si, Si-OH and H_2_O. However, the CH*_x_* stretching band at about 2968 cm^−1^ is more pronounced. The stretching band might correspond to methyl groups attached to silicon (Si–CH_3_). This is corroborated by a pronounced new vibration signal found at 1275 cm^−1^, which can be assigned to the C–H bending in Si–(CH_3_)*_x_* (*x* = 1–3) [26]. The absorption bands at 850 and 800 cm^−1^, corresponding to Si–C and CH_3_ rocking vibrations, support this. A higher number of methyl groups connected to Si is reported to move the position of the Si–(CH_3_)*_x_* bending to smaller wave numbers [41]. Since the observed position at 1275 cm^−1^ is associated with a singular methyl group per Si, the coating seems to be mostly well connected by siloxane bonds.

Furthermore, the introduction of a sample into the vacuum (Figure 7b) resulted in a significantly reduced peak at 1385 cm^−1^, but not in the removal of the CH*_x_* band at 2968 or Si–CH_3_ at 1275 cm^−1^. Hence, it can be assumed that not all of the organic parts of the sample originate from adsorbed by-products. This indicates that, in contrast to the coatings with TEOS, HMDSO-based coatings from this process with air actually contain a significant amount of organic compounds. This opens up interesting applications, e.g., the modification of the surface wettability shown in Section 3.3.1.

**Figure 8:**
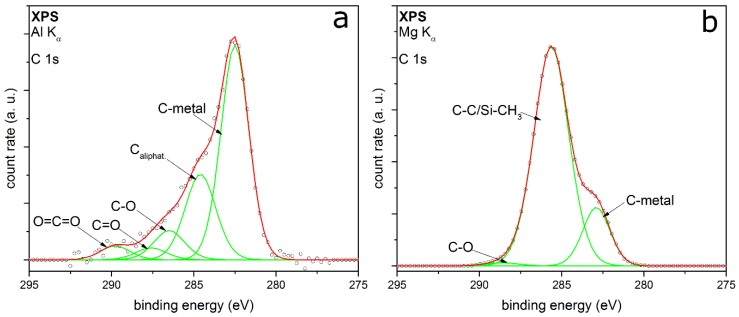
XPS detail spectra C 1s of coated titania particles with TEOS (**a**) and Pt particles with HMDSO as the precursor (**b**).

XPS measurements of the C 1s detail spectra of TEOS and HMDSO coated particles support these assumptions. Figure 8a shows the C 1s peak of the TEOS coated particles. It is fitted with five peaks very well. The two peaks with the highest relative intensities are at E_B_ 282.5 and 284.6 eV, respectively. The first one corresponds to the carbon-metal-bonding from the sample holder [42]. The second peak can be assigned to aliphatic carbon with a relative intensity of 0.25. Further relative intensities are summarized in Table 2.

The other peaks from this core orbital are at E_B_ 286.5 eV, 287.6 eV and 289.7 eV, respectively. They are to be assigned to carbon-oxygen-bonds in different types of bonding, belonging to C=O and to C–O [33]. The third bonding for carbon-oxygen-species is the O=C=O bonding [43]. All in all, the carbon-oxygen-bonds have a relative intensity of 0.18. It can be assumed that there are small aliphatic carbon chains bonding to oxygen. This leads to a mostly hydrophilic character of the organic by-products remaining in the coated layer.

Figure 8b shows the C 1s detail peak of HMDSO coated particles. The intensity of this peak is about 5.5 times higher than the intensity of the TEOS coated particles. This means that there will be much more by-products or remnants of HMDSO on the top or inside the coating. The C 1s peak can only be fitted by three peaks at E_B_ at 282.9, 285.6 and 288.6 eV. They correspond to the carbon-metal-bonding, aliphatic carbon and C–O, whereby the aliphatic carbon has the highest relative intensity. This could indicate organic by-products remaining in the inside of the film or some contamination on the metal particle surface and the coating surface. At the same binding energy, there is the possibility of CH*_x_*–Si bonding, which appears at nearly the same binding energy as the aliphatic carbon [44]. As observed in the FTIR measurements, these organic compounds could consist of small hydrophobic aliphatic carbon chains bonded to silicon (e.g., Si–CH_3_). This is in good agreement with the proposed statements from the two workings groups of Rügner and Alexander. HMDSO does not necessarily react completely to silicon dioxide in an oxygen plasma flow and CH_3_-groups can remain in the deposited layer and form a silicon oxide based plasma polymer [45,46].

**Figure 9:**
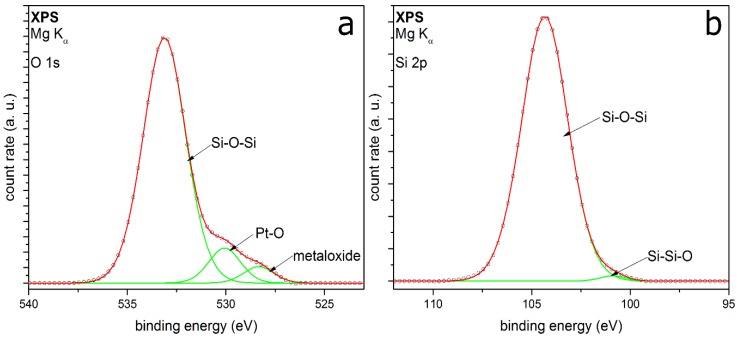
XPS detail spectra O 1s (**a**) and Si 2p (**b**) of coatings on Pt metal particles with HMDSO as the precursor.

Figure 9 shows the O 1s (a) and the Si 2p peak (b) of HMDSO-coated particles. The O 1s is fitted with three peaks at E_B_ at 528.3, 530.0 and 533.1 eV. The first peak belongs to the already known peak from the sample holder. The second peak could be assigned to oxidized platinum metal particles, which have been coated [47]. However, these two metal peaks could be assigned to the typical peak structure of the O 1s from HMDSO deposited films, reported from Kashiwagi et al. [48] as well. The peak with the highest intensity corresponds to Si–O–Si, which fits to the Si 2p results. The peak at 104.3 eV in the Si 2p spectrum shows the highest intensity and belongs to this bonding as well. The second fitted peak conforms to the Si–Si–O bonding. While no CH_3_–Si bonding could be detected, this does not necessarily contradict the previous results. It can be assumed that there might only be one monolayer of silicon carbon bonding on the surface of the coating. The content of this bonding would be too small to be detected beside the high intensity of the silicon oxide bonding.

### 3.3. Application Examples for the Coatings

#### 3.3.1. Modification of Powder Wettability

**Figure 10:**
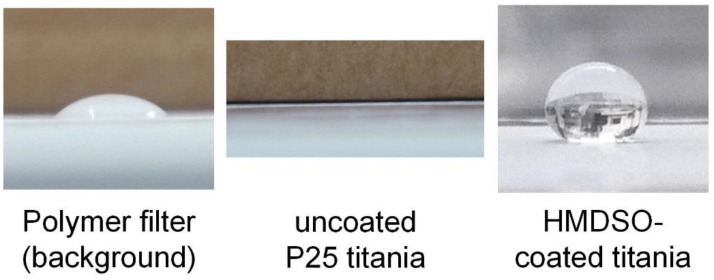
Photographs of water droplets on the filter, on uncoated titania particles on the filter and on HMDSO-coated titania on the filter.

A simple application of the different coatings is the modification of the surface wettability of powders. The inorganic silica coatings from TEOS should be hydrophilic, due to the silanol groups, while the silica-organic coating from HMDSO might be hydrophobic, due to the presence of organic groups. This can be easily checked with a simple sessile drop test, where a droplet of water is placed on the sample and the contact angle is measured. The measurement was done directly on the powder samples collected on a polycarbonate membrane filter, which was hence the background of the measurement. A water droplet on the pristine polymer filter had a contact angle of about 70° (Figure 10, left). As shown in Figure 10 (middle), a water droplet placed on uncoated P25 (after atomization and sampling) instantaneously seeped into the powder, wetting it completely and indicating a high hydrophilicity of the material. The same behavior was observed with uncoated platinum. If the particles were coated with TEOS, the observation was identical, showing that the inorganic coating was indeed hydrophilic. However, a droplet placed on particles coated with HMDSO showed a very different behavior (Figure 10, right). The water was not able to wet the powders, resulting in a contact angle of about 150°. If the sample was tilted, the droplet rolled off. This was observed for both HMDSO-coated P25 and HMDSO-coated Pt particles and shows that the coating can be used to alter the powder surface from hydrophilic to hydrophobic. The hydrophobic character remained after outgassing in the vacuum.

#### 3.3.2. Photocatalysis Effects of TEOS-Coated P25 Titania

**Figure 11:**
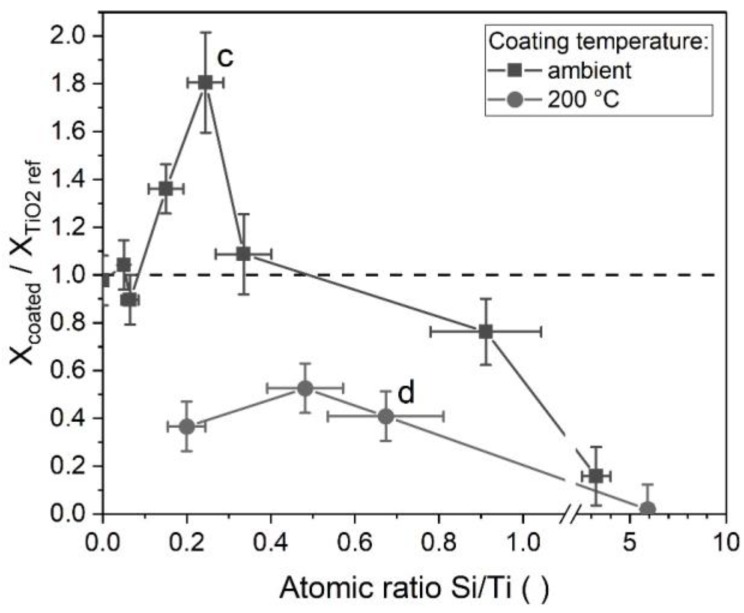
Ratio of the conversions of silica coated titania to reference samples of titania of equal mass in dependence on the coating amount after 1 h under UV radiation. c and d correspond to the respective TEM micrographs in Figure 2.

The photocatalytic degradation of organic compounds by TiO_2_ has been the topic of much dedicated research and is believed to involve the creation of electron hole pairs by absorption of UV light, the formation of radicals on the particle surfaces, the adsorption of organic compounds from the fluid, the reaction and subsequent desorption of products [49,50]. The photocatalytic activity of silica-modified P25 titania was studied as described in Section 2.4 for different coating thicknesses, characterized by the atomic ratio of Si/Ti in the sample (from EDX). The coatings were applied either with the TEOS process at ambient temperature or at 200 °C. The coating amount on the particles was controlled by the initial TEOS concentration and all other parameters were kept constant. The aerosol residence time was 4.2 min at ambient temperature or 4 s at 200 °C. The results are shown in Figure 11 as the ratio of the conversion of the coated sample to the conversion of the respective uncoated reference. Since a thicker coating equals a lower effective mass of titania in a sample in comparison to the reference, the results were mass corrected with the mass fraction of titania, which was determined with EDX. A ratio above one in Figure 11 shows an improvement of the photocatalytic activity of the sample in comparison to the uncoated reference, while a ratio below one signifies a decreased activity.

In many applications, titania particles are coated with silica to suppress their photocatalytic behavior, e.g., for use in sunscreens. Here, the coating acts as a barrier between the photogenerated species in the titania and the organic compounds in the surrounding fluid. Hence, one would expect a decrease of the conversion ratio with increasing coating amount, since the coating should cover an increasingly larger fraction of the titania surfaces. For the coatings acquired at 200 °C, this seems to be the case. All data points show a clear decrease in activity, indicating a passivation by the coatings. Even relatively small coating amounts showed an inhibiting effect. However, the particles coated at ambient temperature behaved differently. While their activity decreases at higher coating amounts (approx. above 0.5 Si/Ti), a significant increase was observed beforehand. This seems to indicate that the coatings improve the photocatalytic decomposition of MB.

As described in Section 3.1, differences in the coating morphologies between those acquired at 24 °C and those at 200 °C were found. The particles shown in Figure 2c,d correspond to the marked data points in Figure 11. As discussed before, the coatings from elevated temperatures covered the titania surfaces more homogeneously, while the coatings from ambient temperatures collected primarily in the inner parts of the aggregates. This indicates that the results might in part be an effect of the degree of surface coverage, with a higher coverage decreasing the activity. At high coating amounts, the inhomogeneous coatings (24 °C) will cover all surfaces and reduce the activity, while the more homogeneous coatings (200 °C) require smaller silica amounts for the same effect.

However, this cannot explain the increase in the activity at low coating amounts at 24 °C, which shows that additional effects must play a role. While the improvement of titania photocatalysts by silica has been observed before, the literature offers a number of different explanations. Both Hu et al. [51] and Gholami et al. [52] explained their findings with a reduced recombination rate of photogenerated electrons and holes, which corresponds to a higher photocatalytic activity. Another possible explanation could be the improved adsorption and desorption behavior of MB on the particles with coatings. In the here presented work no difference in MB concentration for either coated or reference particles after 1 h of dark adsorption was found. However, it is known from the literature that “inert” surfaces close to the photocatalytic ones can positively influence the performance of a catalyst [53] and even very thin, but complete, coatings were shown to improve the photocatalytic degradation of adsorbed aromatic compounds [54]. In particular, the coatings might improve the adsorption rate of reactants or the desorption of products. Kim and Ehrman found an improvement of the photocatalytic activity by bridge-shaped coatings in interparticle connections, which is a similar morphology as that observed here for the coating at 24 °C as shown in Figure 2c [55]. Finally, it is known that TiO_2_-SiO_2_ mixed oxides can exhibit an improved photocatalytic activity in comparison to pure TiO_2_, due to a modification of the electronic structure at the interface [56]. Such oxides can be formed in thin coatings. As shown before (Section 3.2), mixed oxides might be present in the coated samples. While a conclusive explanation of the observed behavior is beyond the scope of this work, the results are interesting and might warrant further research.

## 4. Conclusions

A plasma-enhanced CVD coating process with air was used to produce core-shell particles in a post-discharge environment of a DBD. Two different coating precursors, TEOS and HMDSO, were used at different temperatures during coating. Differences in the coating morphologies were visible in TEM micrographs for the different combinations of core material, precursor and temperature. While TEOS on Pt or Au resulted in mostly homogeneous coatings, HMDSO formed eccentric coatings on these particles. For titania particles coated with TEOS at 24 °C, much of the coating material was collected in the inner parts of the aggregates. The comparison of the chemical composition of the coatings with FTIR, EDX and XPS showed that the use of TEOS resulted in mostly inorganic silica coatings with some adsorbed by-products, while HMDSO led to silica-organic films. Moreover, the core particles showed an influence on the coating composition, with the possible formation of mixed oxides in coated titania. The coatings were used to demonstrate simple applications such as the formation of hydrophobic particle surfaces with HMDSO and the improvement or inhibition of the photoactivity of P25 titania with TEOS.

## Figures and Tables

**Table 1:** Summarized XPS results from the particles coated with TEOS as precursor.

System	Detail Peak	Binding Energy (eV)	FWHM (eV)	Relative Intensity
TEOS/Au	O 1s	530.3	1.1	0.01
		532.3	1.3	0.03
		532.7	2.2	0.61
		533.6	2.2	0.30
		535.4	2.2	0.05
	Si 2p	100.6	2.5	0.04
		103.7	2.5	0.29
		104.9	2.5	0.68
TEOS/TiO_2_	O 1s	528.5	1.8	0.03
		530.3	2.2	0.14
		532.4	2.2	0.16
		532.8	2.2	0.30
		533.0	1.8	0.33
		535.0	2.0	0.03
	Si 2p	100.7	2.1	0.06
		102.5	2.1	0.93

**Table 2:** Summarized XPS results from Pt metal particles coated with HMDSO and from the C 1s from TEOS-coated titania.

System	Detail Peak	Binding Energy (eV)	FWHM (eV)	Relative Intensity
HMDSO/Metal	O 1s	528.3	1.8	0.04
		530.0	1.8	0.09
		533.1	2.5	0.87
	Si 2p	100.8	1.5	0.01
		104.3	2.7	0.99
	C 1s	282.9	1.9	0.17
		285.6	2.5	0.81
		288.6	2.3	0.01
TEOS/TiO_2_	C 1s	282.5	2.0	0.58
		284.6	2.2	0.25
		286.5	2.2	0.09
		287.6	2.2	0.04
		289.7	2.1	0.04

## References

[1] Ghosh Chaudhuri R., Paria S. (2012). Core/Shell Nanoparticles: Classes, Properties, Synthesis Mechanisms, Characterization, and Applications. Chem. Rev..

[2] Egerton T. (1998). The Modification of Fine Powders by Inorganic Coatings. KONA Powder Part. J..

[3] King D.M., Liang X., Burton B.B., Kamal Akhtar M., Weimer A.W. (2008). Passivation of pigment-grade TiO_2_ particles by nanothick atomic layer deposited SiO_2_ films. Nanotechnology.

[4] Croissant J.G., Fatieiev Y., Almalik A., Khashab N.M. (2018). Mesoporous Silica and Organosilica Nanoparticles: Physical Chemistry, Biosafety, Delivery Strategies, and Biomedical Applications. Adv. Healthc. Mater..

[5] Kruis F.E., Fissan H., Peled A. (1998). Synthesis of nanoparticles in the gas phase for electronic, optical and magnetic applications—A review. J. Aerosol Sci..

[6] Teleki A., Heine M.C., Krumeich F., Akhtar M.K., Pratsinis S.E. (2008). In Situ Coating of Flame-Made TiO_2_ Particles with Nanothin SiO_2_ Films. Langmuir.

[7] Qi F., Moiseev A., Deubener J., Weber A. (2011). Thermostable photocatalytically active TiO_2_ anatase nanoparticles. J. Nanoparticle Res..

[8] George S.M. (2010). Atomic Layer Deposition: An Overview. Chem. Rev..

[9] Powell Q.H., Fotou G.P., Kodas T.T., Anderson B.M., Guo Y. (1997). Gas-phase coating of TiO_2_ with SiO_2_ in a continuous flow hot-wall aerosol reactor. J. Mater. Res..

[10] Kogelschatz U. (2003). Dielectric-barrier discharges: Their history, discharge physics, and industrial applications. Plasma Chem. Plasma Process..

[11] Vons V., Creyghton Y., Schmidt-Ott A. (2006). Nanoparticle production using atmospheric pressure cold plasma. J. Nanoparticle Res..

[12] Nessim C., Boulos M., Kogelschatz U. (2009). In-flight coating of nanoparticles in atmospheric-pressure DBD torch plasmas. Eur. Phys. J. Appl. Phys..

[13] Post P., Jidenko N., Weber A.P., Borra J.-P. (2016). Post-Plasma SiO_x_ Coatings of Metal and Metal Oxide Nanoparticles for Enhanced Thermal Stability and Tunable Photoactivity Applications. Nanomaterials.

[14] Post P., Weber A.P. (2018). Beschichtung von gasgetragenen Nanopartikeln mit SiO_2_ mithilfe eines plasma-unterstützten CVD-Prozesses bei Umgebungsbedingungen. Chem. Ing. Tech..

[15] Adachi M., Okuyama K., Tohge N., Shimada M., Sato J., Muroyama M. (1993). Particle Generation and Film Formation in an Atmospheric-Pressure Chemical Vapor Deposition Reactor Using the Tetraethylorthosilicate (TEOS)/He, TEOS/O_2_/He, and TEOS/O_3_/He Systems. Jpn. J. Appl. Phys..

[16] Romet S., Couturier M.F., Whidden T.K. (2001). Modeling of silicon dioxide chemical vapor deposition from tetraethoxysilane and ozone. J. Electrochem. Soc..

[17] Tabrizi N.S., Ullmann M., Vons V.A., Lafont U., Schmidt-Ott A. (2009). Generation of nanoparticles by spark discharge. J. Nanoparticle Res..

[18] Evonik Industries AEROXIDE, AERODISP and AEROPERL Titanium Dioxide as Photocatalyst. http://www.aerosil.com/sites/lists/RE/DocumentsSI/TI-1243-Titanium-Dioxide-as-Photocatalyst-EN.pdf.

[19] Krischok S., Höfft O., Günster J., Stultz J., Goodman D., Kempter V. (2001). H_2_O interaction with bare and Li-precovered TiO_2_: Studies with electron spectroscopies (MIES and UPS (HeI and II)). Surf. Sci..

[20] Klarhöfer L., Roos B., Viöl W., Höfft O., Dieckhoff S., Kempter V., Maus-Friedrichs W. (2008). Valence band spectroscopy on lignin. Holzforschung.

[21] Heinlin J., Isbary G., Stolz W., Morfill G., Landthaler M., Shimizu T., Steffes B., Nosenko T., Zimmermann J., Karrer S. (2011). Plasma applications in medicine with a special focus on dermatology: Plasma medicine. J. Eur. Acad. Dermatol. Venereol..

[22] Shirley D.A. (1972). High-Resolution X-Ray Photoemission Spectrum of the Valence Bands of Gold. Phys. Rev. B.

[23] Scofield J.H. (1976). Hartree-Slater subshell photoionization cross-sections at 1254 and 1487 eV. J. Electron Spectrosc. Relat. Phenom..

[24] Yeh J.J., Lindau I. (1985). Atomic subshell photoionization cross sections and asymmetry parameters: 1 ⩽ Z. ⩽ 103. At. Data Nucl. Data Tables.

[25] Bewig K.W., Zisman W.A. (1965). The wetting of gold and platinum by water. J. Phys. Chem..

[26] Moravej M., Hicks R.F. (2005). Atmospheric Plasma Deposition of Coatings Using a Capacitive Discharge Source. Chem. Vap. Depos..

[27] Klapiszewski Ł., Siwińska-Stefańska K., Kołodyńska D. (2017). Preparation and characterization of novel TiO_2_/lignin and TiO_2_-SiO_2_/lignin hybrids and their use as functional biosorbents for Pb (II). Chem. Eng. J..

[28] Himpsel F.J., McFeely F.R., Taleb-Ibrahimi A., Yarmoff J.A., Hollinger G. (1988). Microscopic structure of the SiO_2_/Si interface. Phys. Rev. B.

[29] Hollinger G., Himpsel F.J. (1984). Probing the transition layer at the SiO_2_-Si interface using core level photoemission. Appl. Phys. Lett..

[30] Cerofolini G.F., Galati C., Renna L. (2002). Accounting for anomalous oxidation states of silicon at the Si/SiO_2_ interface. Surf. Interface Anal..

[31] McCafferty E., Wightman J.P. (1998). Determination of the concentration of surface hydroxyl groups on metal oxide films by a quantitative XPS method. Surf. Interface Anal..

[32] D’Souza A.S., Pantano C.G. (2004). Mechanisms for Silanol Formation on Amorphous Silica Fracture Surfaces. J. Am. Ceram. Soc..

[33] Gustus R., Gruber W., Wegewitz L., Geckle U., Prang R., Kübel C., Schmidt H., Maus-Friedrichs W. (2014). Decomposition of amorphous Si2C by thermal annealing. Thin Solid Films.

[34] Bebensee F., Voigts F., Maus-Friedrichs W. (2008). The adsorption of oxygen and water on Ca and CaO films studied with MIES, UPS and XPS. Surf. Sci..

[35] Barr T.L. (1983). An XPS study of Si as it occurs in adsorbents, catalysts, and thin films. Appl. Surf. Sci..

[36] Anwar M., Hogarth C.A., Bulpett R. (1990). An XPS study of amorphous MoO_3_/SiO films deposited by co-evaporation. J. Mater. Sci..

[37] Dahle S., Wegewitz L., Qi F., Weber A.P., Maus-Friedrichs W. (2013). Silicon Dioxide Coating of Titanium Dioxide Nanoparticles from Dielectric Barrier Discharge in a Gaseous Mixture of Silane and Nitrogen. Plasma Chem. Plasma Process..

[38] Cheung S.H., Nachimuthu P., Joly A.G., Engelhard M.H., Bowman M.K., Chambers S.A. (2007). N incorporation and electronic structure in N-doped TiO_2_(110) rutile. Surf. Sci..

[39] Kaufmann E.N., Kaufmann E.N. (2012). Common Concepts in Materials Characterization, Introduction. Characterization of Materials.

[40] Chinh V.D., Broggi A., Di Palma L., Scarsella M., Speranza G., Vilardi G., Thang P.N. (2018). XPS Spectra Analysis of Ti^2+^, Ti^3+^ Ions and Dye Photodegradation Evaluation of Titania-Silica Mixed Oxide Nanoparticles. J. Electron. Mater..

[41] Fanelli F., Lovascio S., d’Agostino R., Arefi-Khonsari F., Fracassi F. (2010). Ar/HMDSO/O2 Fed Atmospheric Pressure DBDs: Thin Film Deposition and GC-MS Investigation of By-Products. Plasma Process. Polym..

[42] Ramqvist L. (1969). Charge transfer in transition metal carbides and related compounds studied by ESCA. J. Phys. Chem. Solids.

[43] Ichihara T., Aizawa K. (1998). 1/f noise in a-Si_1–*x*_C*_x_*: H thin films as novel thermistor materials for micro-machined IR sensors. J. Non. Cryst. Solids.

[44] Alexander M.R., Jones F.R., Short R.D. (1997). Mass spectral investigation of the radio-frequency plasma deposition of Hexamethyldisiloxane. J. Phys. Chem. B.

[45] Rügner K., Reuter R., Ellerweg D., de los Arcos T., von Keudell A., Benedikt J. (2013). Insight into the Reaction Scheme of SiO_2_ Film Deposition at Atmospheric Pressure: Insight into the Reaction Scheme of SiO_2_ Film Deposition. Plasma Process. Polym..

[46] Alexander M.R., Short R.D., Jones F.R., Stollenwerk M., Zabold J., Michaeli W. (1996). An X-ray photoelectron spectroscopic investigation into the chemical structure of deposits formed from hexamethyldisiloxane/ oxygen plasmas. J. Mater. Sci..

[47] Shukla A., Neergat M., Bera P., Jayaram V., Hegde M. (2001). An XPS study on binary and ternary alloys of transition metals with platinized carbon and its bearing upon oxygen electroreduction in direct methanol fuel cells. J. Electroanal. Chem..

[48] Kashiwagi K., Yoshida Y., Murayama Y. (1991). Hybrid Films Formed from Hexamethyldisiloxane and SiO by Plasma Process. Jpn. J. Appl. Phys..

[49] Lee S.-Y., Park S.-J. (2013). TiO_2_ photocatalyst for water treatment applications. J. Ind. Eng. Chem..

[50] Nakata K., Fujishima A. (2012). TiO_2_ photocatalysis: Design and applications. J. Photochem. Photobiol. C Photochem. Rev..

[51] Hu S., Li F., Fan Z. (2012). Preparation of SiO_2_-Coated TiO_2_ Composite Materials with Enhanced Photocatalytic Activity Under UV Light. Bull. Korean Chem. Soc..

[52] Gholami T., Bazarganipour M., Salavati-Niasari M., Bagheri S. (2015). Photocatalytic degradation of methylene blue on TiO_2_@SiO_2_ core/shell nanoparticles: Synthesis and characterization. J. Mater. Sci. Mater. Electron..

[53] Minero C., Catozzo F., Pelizzetti E. (1992). Role of adsorption in photocatalyzed reactions of organic molecules in aqueous titania suspensions. Langmuir.

[54] Nussbaum M., Paz Y. (2012). Ultra-thin SiO_2_ layers on TiO_2_: Improved photocatalysis by enhancing products’ desorption. Phys. Chem. Chem. Phys..

[55] Kim S., Ehrman S.H. (2009). Photocatalytic activity of a surface-modified anatase and rutile titania nanoparticle mixture. J. Colloid Interface Sci..

[56] Gao X., Wachs I.E. (1999). Titania–silica as catalysts: Molecular structural characteristics and physico-chemical properties. Catal. Today.

